# Biosynthesis and regulation of terpenoids from basidiomycetes: exploration of new research

**DOI:** 10.1186/s13568-021-01304-7

**Published:** 2021-11-15

**Authors:** Qi Wang, Rui Cao, Yuna Zhang, Pengyan Qi, Lizhi Wang, Shiming Fang

**Affiliations:** 1 School of Chinese Materia Medica, Tianjin University of Traditional Chinese Medicine, Tianjin, 301617 China; 2 Tianjin State Key Laboratory of Modern Chinese Medicine, Tianjin University of Traditional Chinese Medicine, Tianjin, 301617 China

**Keywords:** Basidiomycetes, Terpenoids, Biosynthesis, CYP450

## Abstract

Basidiomycetes, also known as club fungi, consist of a specific group of fungi. Basidiomycetes produce a large number of secondary metabolites, of which sesquiterpenoids, diterpenoids and triterpenoids are the primary components. However, these terpenoids tend to be present in low amounts, which makes it difficult to meet application requirements. Terpenoid biosynthesis improves the quantity of these secondary metabolites. However, current understanding of the biosynthetic mechanism of terpenoids in basidiomycetes is insufficient. Therefore, this article reviews the latest research on the biosynthesis of terpenoids in basidiomycetes and summarizes the CYP450 involved in the biosynthesis of terpenoids in basidiomycetes. We also propose opportunities and challenges for chassis microbial heterologous production of terpenoids in basidiomycetes and provide a reference basis for the better development of basidiomycete engineering.

## Key points

- The biosynthetic pathways of the primary products of basidiomycetes are reviewed.
- The biosynthesis of sesquiterpenes and triterpenoids is described in detail.
- A summary of information concerning the influence of CYP450 on the biosynthesis of basidiomycetes is provided*.*

## Introduction

Basidiomycetes and ascomycetes are the two most important phyla in the fungal kingdom (Lin et al. 2019). For thousands of years, many basidiomycetes, such as *Lentinus edodes*, *Auricularia auricula* and *Hericium erinaceus*, have been used as food (Lei et al. 2019; Zhao et al. 2020; Miao et al. 2020; Hu et al. 2020). Additionally, certain fungi, such as *Ganoderma lucidum* and *Poria cocos* (Lin et al. 2020; Wang et al. 2020a, b, c), have been used as medicinal plants. Basidiomycetes, particularly their sporocarps, contain abundant natural products (Tian et al. 2020). For example, pleuromutilin, a diterpenoid natural product, shows moderate activities against gram-positive bacteria and mycoplasmas (Zhang et al. 2021a, b, c). Due to its low cytotoxicity in mammals and low environmental impact, pleuromutilin has been gradually developed into a commercial antibiotic. Pleuromutilin is derived from certain types of fungi and is widely used to produce antibiotics from various foods via deep fermentation. However, fermentation efficiency is affected by many factors. Due to the high cost of fermentation and high energy consumption, the traditional method of producing pleuromutilin is not suitable for industrial mass production. In recent years, many scholars have been actively studying related processes to improve the yield of pleuromutilin (Sun et al. 2017). White rot fungi have long been used as effective degradation tools for environmental pollutants (Jureczko et al. 2021) and contain abundant terpenoids. For example, *Hypsizygus marmoreus* contains antitumour components, terpene synthases hypsin and hypsiziprenol (Min et al. 2018), and GA contains anticancer triterpenes from *G. lucidum* (Guo et al. 2020). However, because the growth of white rot fungi requires strong lighting and many nutrients (Pawlik et al. 2020), large-scale cultivation is difficult. In addition, the strict requirements of some basidiomycetes for growth conditions also limit their development and use (Lu et al. 2020).

**Fig. 1:**
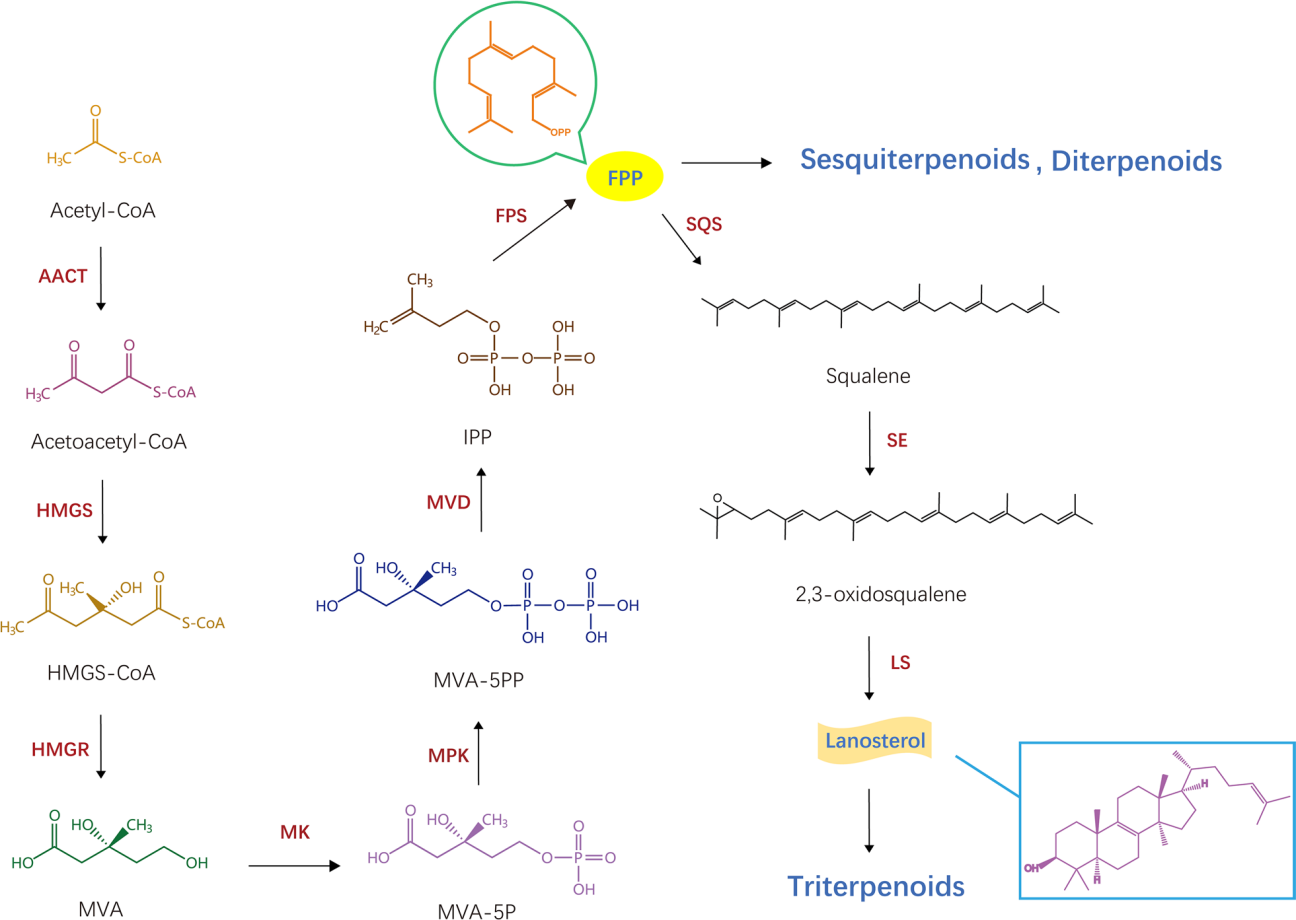
Biosynthetic pathway of terpenoids in basidiomycetes

Terpenoids are currently the largest group of natural products in fungi (Quin et al. 2014). However, compared with ascomycetes, sesquiterpenes, diterpenoids and triterpenoids are the primary components in basidiomycetes. Due to the complex chemical structure of terpenoid metabolites, they are difficult to synthesize chemically (Xiao and Zhong 2016). Therefore, the method of obtaining terpenoids by biosynthesis has attracted increasing attention to meet the large needs of clinical applications and large-scale industrial production. In nature, there are two primary biosynthetic pathways: the mevalonate (MVA) pathway and the methylphenidate (MEP) pathway (Liao et al. 2016). The terpenoids in basidiomycetes are primarily synthesized through the mevalonate (MVA) pathway (Perez-Gil and Rodriguez-Concepcion 2013). The MVA pathway begins with acetyl-CoA, which is catalysed by several enzymes (AACT, HMGS, HMGS-CoA, HMGR) to generate mevalonate (MVA), of which HMGR is the recognized rate-limiting enzyme (Costa et al. 2019). Subsequently, isopentenyl pyrophosphate (IPP) is formed by the catalysis of MK, MPK, MVD and other enzymes (Hu et al. 2019a, b). IPP is a precursor intermediate metabolite of terpenoid biosynthesis. IPP and DMAPP generate FPP and GGPP under the catalysis of FPS and GGPPS, respectively, which are precursors of sesquiterpenes and diterpenes, respectively (Xiao and Zhong 2016; Ding et al. 2014). Conversely, FPP generates lanosterol under the catalysis of SQS, SE and LS. Lanosterol is the precursor of triterpenoids and is the key to the synthesis of triterpenes and sterols. Lanosterol can generate a variety of triterpenoids with a skeleton through a series of chemical reactions (Shi et al. 2010; Zhu et al. 2019) (Fig. 1).

Many studies have described the biosynthesis of secondary metabolites from plants, but the application of enzyme mechanism biosynthesis of terpenoids in basidiomycetes is still in its early stages. As one of the largest natural products of basidiomycetes, terpenoids have great research value and potential. However, only several terpenoid has been reported previously: triterpenoid ganoderic acid (GA) and diterpenoid pleuromutilin. This study systematically summarized the terpenoids in basidiomycetes via synthetic biology, focusing on sesquiterpenoids, triterpenoids and CYP450. This review is dedicated to describing all that is currently known about basidiomycetes.

### Biosynthesis of sesquiterpenoids

#### Identification and expression of genes related to sesquiterpene biosynthesis in basidiomycetes

Basidiomycetes are rich sources of sesquiterpene compounds. A variety of biologically activated sesquiterpene compounds have been identified from basidiomycetes. For example, *Inonotus obliquus* (Chaga), a basidiomycete derived from Hymenochaetales, commonly appears in the form of irregular sclerotia in nature and can produce a variety of bioactive terpene compounds with antitumour and anti-inflammatory effects. Among the terpenes found in Chaga, sesquiterpenes (bergamotene, selinene, and santalene) and triterpenes (betulin, betulinic acid, lanosterol, inotodiol, and trametenolic acid) have been identified (Fradj et al. 2019). *Polyporus brumalis*, a white-rot fungus of basidiomycetes, has been shown to synthesize sesquiterpenes using a single carbon source in a liquid medium (Lee et al. 2017). According to reports, differentially expressed genes related to terpene metabolism in *P. bumbellatus* were identified by NGS technology. Sequencing results identified 25,000 single genes and 127 metabolic pathways, in which sesquiterpenes β-eudesmane and β-eudesmol were only produced in the mycelia of *P. bumbellatus* on the modified medium. After further analysis of samples from the modified medium, results showed that eight single genes involved in the mevalonate (MVA) and methylphenidate (MEP) biosynthetic pathways were significantly upregulated, and germacrene A synthase encoding FPP cyclization was found to be differentially expressed only in the hyphae of the modified medium (Lee et al. 2016). The results of this experiment provide resources for the biosynthesis of sesquiterpenes and the molecular mechanism of terpene metabolism.

**Fig. 2:**
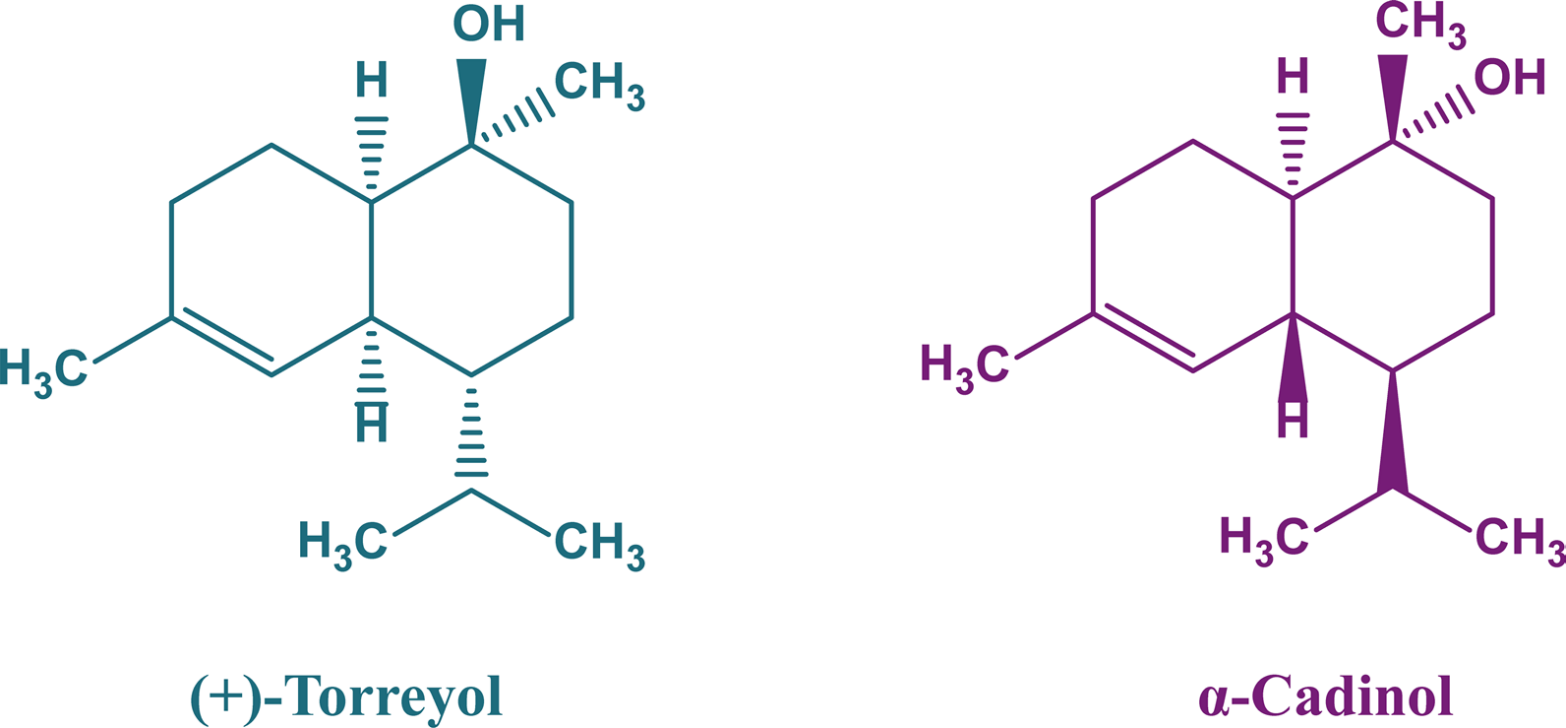
Chemical structures of two cytotoxic sesquiterpene products from *L. rhinocerotis*

Basidiomycete sesquiterpene synthase (STS) has been shown to be easily expressed in heterologous hosts *of E. coli* and *S. cerevisiae* (Wawrzyn et al. 2012; Zelena et al. 2012; Scholtmeijer et al. 2014)*.* A study that sequenced the genome of *Lignosus rhinocerotis* (Cook) RYvarden showed that there were 12 STS genes in *L. rhinocerotis*, while transcriptome studies showed that seven of the 12 STS genes were highly expressed in sclerotium (Yap et al. 2014). Researchers cloned several nuclear-expressed STS genes from *L. rhinocerotis* and expressed them heterologously in *S. cerevisiae*. The products were identified by GC–MS, and two major sesquiterpene products were isolated and characterized. The high expression of three terpene synthase genes in sclerotium proved that the sesquiterpene biosynthesis genes GME3638 and GME3634 (GenBank Accession Numbers: KX281943, KX281944) were involved in the biosynthesis of toreyol and α-cadinol, respectively (Fig. 2). Both (+)-Torreyol and low-activity α-cadinol showed potent cytotoxicity against MCF7 cells, the first reports of pure biological activities of the two sesquiterpenes (Yap et al. 2017).

In addition, 12 sesquiterpenes have been isolated and identified from the fermentation culture of *Sanghuangporus* by NMR spectroscopy, high-resolution mass spectrometry, and other spectroscopies, and showed antibacterial activity against *Bacillus subtilis* (Cheng et al. 2019). Because most sesquiterpene compounds are volatile substances, chemical synthesis methods are difficult to achieve. Therefore, the synthetic biological heterologous expression method markedly reduces the pressure on the sesquiterpene industry and is convenient for medical treatments and industrial production.

#### Terpene synthase- and terpenoid-modifying enzymes lead to the diversity of sesquiterpenes

The structural diversity of sesquiterpenoids is accomplished by the combined action of sesquiterpene synthase (STS) and terpenoid modifying enzymes (e.g., cytochrome P450 monooxygenase (P450s)). In the early stages of biosynthesis, STS plays a key role in the diversification of the backbone structure of sesquiterpenoids by catalysing the highly complex cyclization of the common precursor farnesyl (Weitzel and Simonsen 2013).

**Fig. 3:**
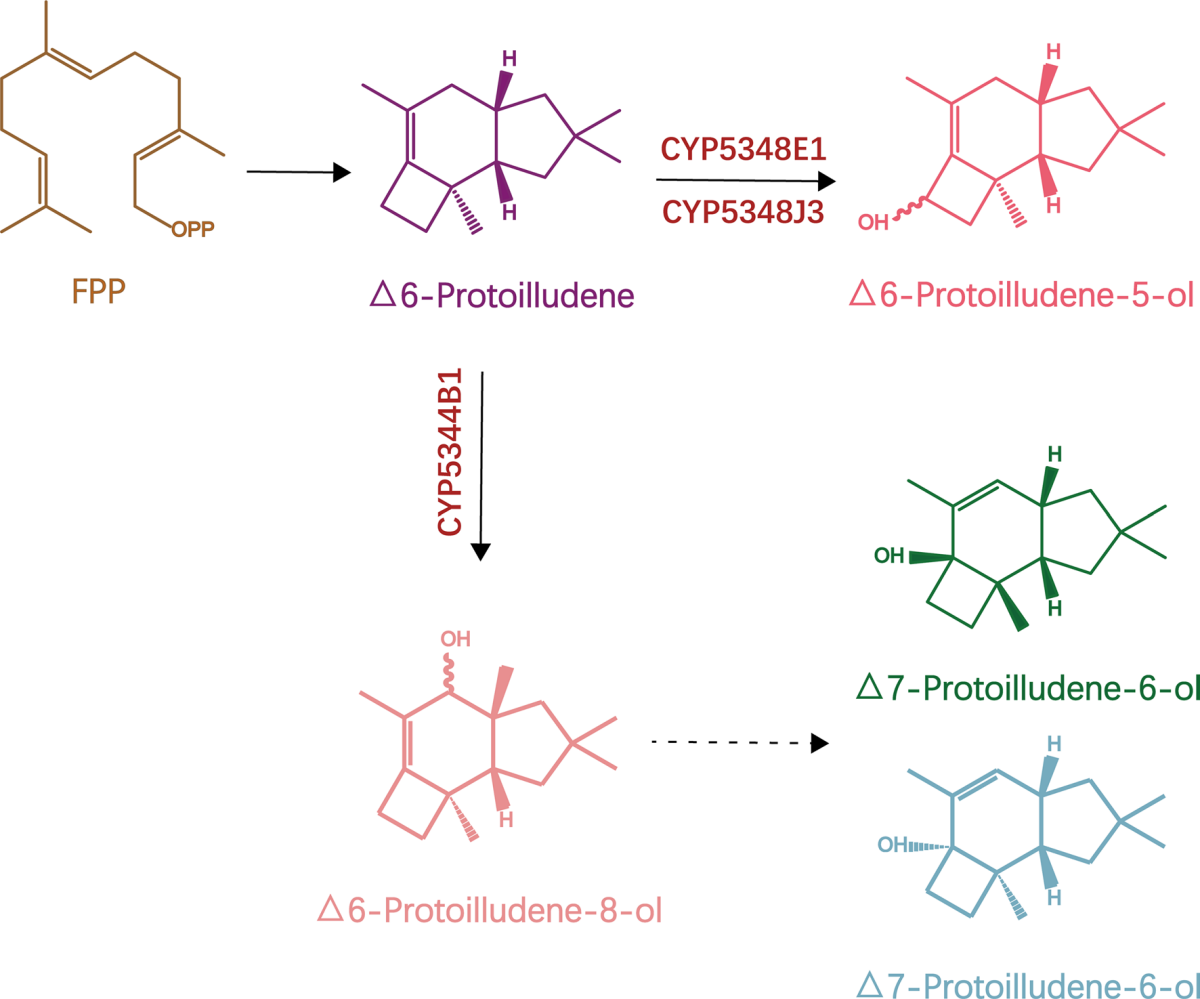
Reaction pathways of protoilludene metabolism by PpSTS and PpCYPs

Studies have isolated 16 sesquiterpene synthase genes from brown-rot basidiomycete *Postia placenta*. The results of heterologous expression in yeast showed that sesquiterpene synthase can produce a series of sesquiterpene scaffolds with different metabolic properties. This experiment was the first to characterize the protoilludene synthase of brown rot basidiomycetes and to perform functional screening of *P. placenta* P450s. Results showed that the coexpression of protoilludene synthase and 184 P450 subtypes can recognize CYP5344B1, CYP5348E1 and CYP5348J3, thereby catalysing the hydroxylation reaction of Δ6-protoilludene to produce Δ6-protoilludene-8-ol and Δ6-protoilludene-5-ol. In addition, by culturing Δ6-protoilludene-8-ol in an acidic medium, an isomer of Δ7-protoilludene-6-ol was obtained (Ichinose and Kitaoka 2018) (Fig. 3).

This experiment identified protoilludene synthase from brown-rot basidiomycetes for the first time, demonstrating the metabolic potential of *P. placenta* to produce sesquiterpenoids and clarifying the biosynthetic mechanism involved in the metabolism of Δ6-protoilludene. In addition, PpCYPs was shown to play an important role in the diversity of *P. placenta* protoilludane-type sesquiterpenoids. The information disclosed in the functional omics research of STS and P450 in this report should paved the way for advanced fungal biology and biotechnology.

**Fig. 4:**
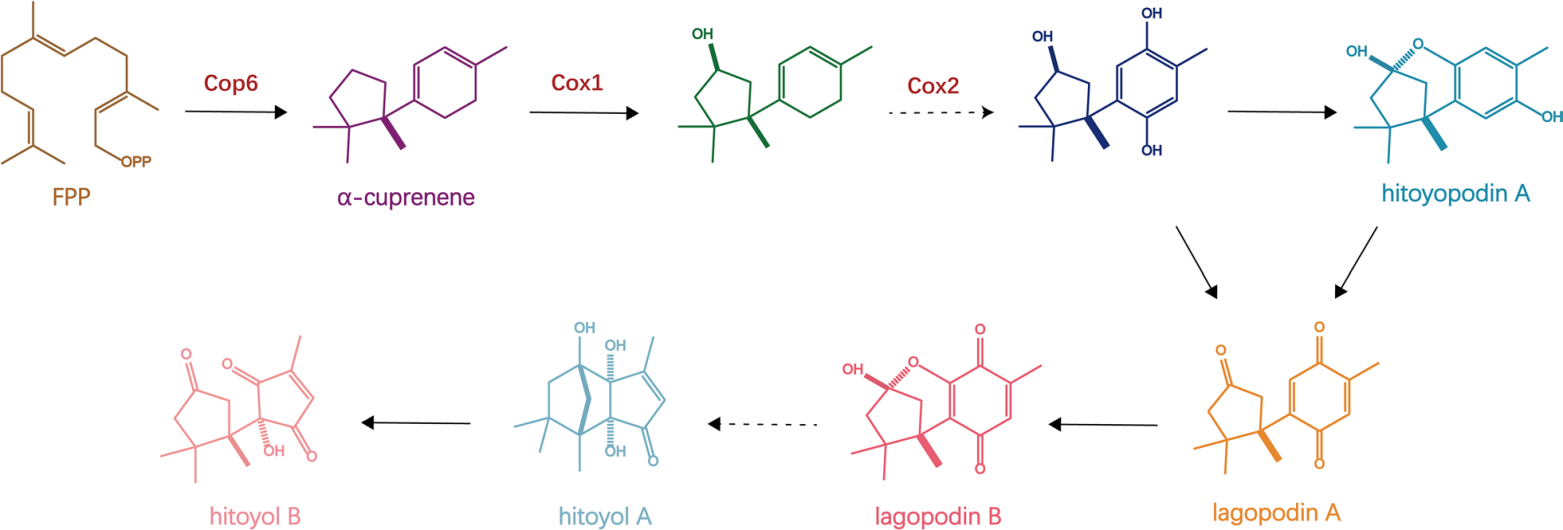
The speculated biosynthetic pathways of lagopodins and hitoyols synthesized through copperene and other intermediates

Lagopodins are natural terpenoid products that are isolated from *Coprinopsis cinerea* and have antibacterial activity against *Staphylococcus aureus*. This series of compounds has a unique sesquiterpene structure, consisting of a five-membered ring and a six-membered ring. Due to their unique chemical structure and potential useful biological activity, lagopodins have gained wide interest in the fields of natural product chemistry, medicinal chemistry and chemical biology (Lagoutte and Winssinger 2017). Analysis of the lagopodin B biosynthetic gene cluster showed that it was produced by the cyclization and oxidation of the terpene cyclase encoded by cop6 and the two cytochrome P450s encoded by cox1 and cox2. Specifically, the biosynthetic pathway of lagopodin B begins with the cyclization of farnesyl pyrophosphate to α-cuprenene under the catalysis of Cop6, which has a highly specific catalytic effect on the synthesis of α-cuprenene (Agger et al. 2009) (Fig. 4).

In this study, the production of lagopodin B and related pathway products increased by overexpressing the terpene cyclase gene cop6 in *C. cinerea* to determine the details of the complex biosynthetic pathways of lagopodin and hitoyol. Random integration of cop6 into the genome of the ku70-deficient *C. cinerea* strain resulted in an approximately 2.4-fold increase in the production of lagopodin B. However, the integration of cop6 into a highly transcribed position within the designated expression promoting region (EBA) chromosome resulted in an approximately 14-fold increase in the production of lagopodin B. This discovery expands the understanding of the biosynthetic pathway of lagopodin–hitoyol (Asai et al. 2020). Although this experiment did not directly prove that the placement of cop6 in EBA led to an increase in gene expression, it successfully increased the product yield, which indicates that the use of EBA may be able to markedly increase the production of poorly biosynthetic target compounds in basidiomycetes.

**Fig. 5:**
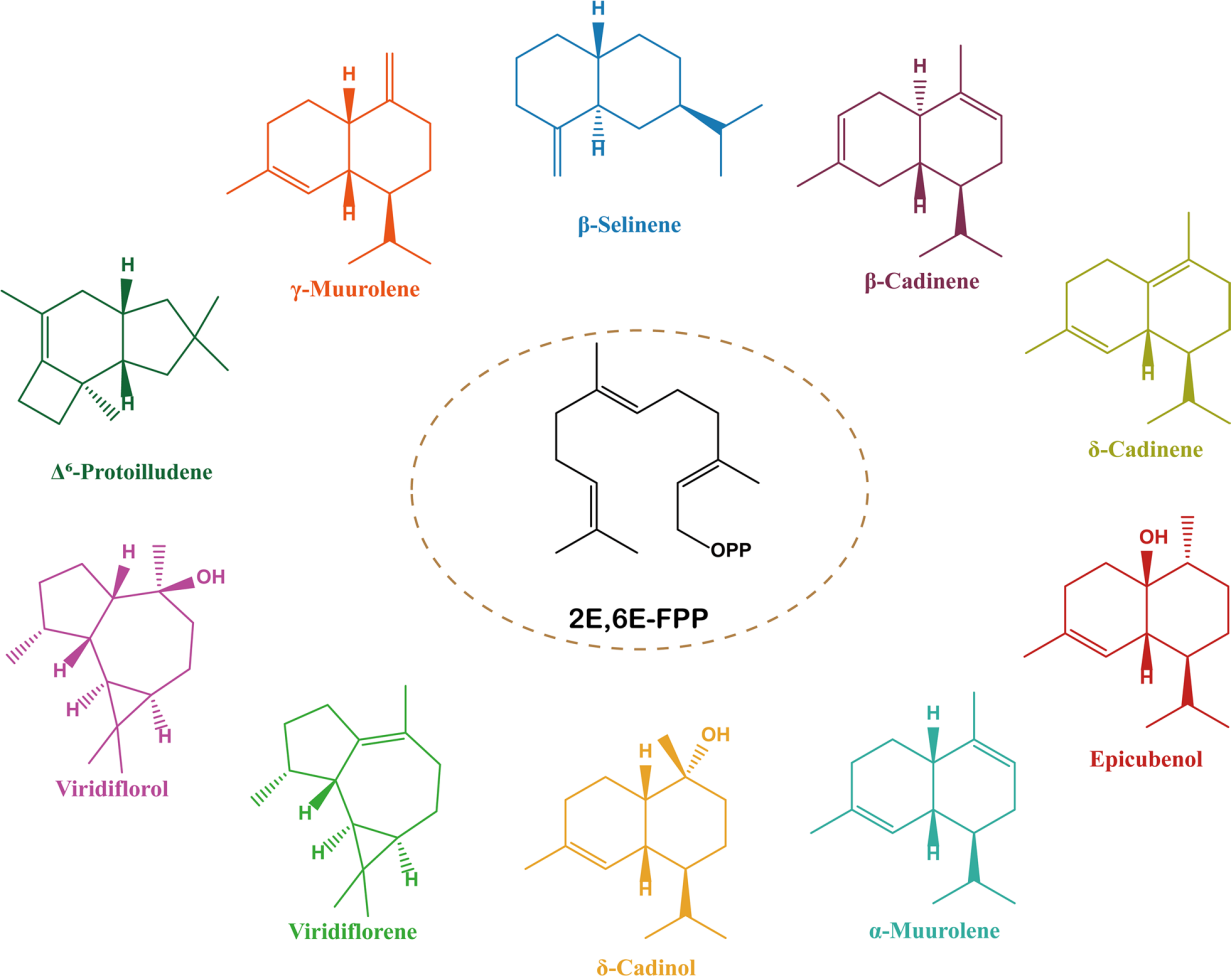
Terpenes produced by *E. coli* expressing STS genes from *A. aegerita*

Eleven putative STSs were also identified in the genome of *Agrocybe aegerita*. These predicted STSs were cloned into the *E. coli* PET vector after codon optimization and transformed into the *E. coli* BL21(DE3) strain. Nine of them are functional (Table 1), and one or more sesquiterpenes can be produced in their liquid cultures (Fig. 5), including two new synthases producing viridiflorol and viridiflorene with antibacterial activity (Zhang et al. 2020). This research provides a basic prediction framework for the discovery of fungal STSs and the biosynthesis of new terpenoids.

**Table 1:** Gene coding for TPS in *A. aegerita*

TPS	ID^a^	Accession number	Gene start	Gene stop	Gene length	Protein length
Agr1	06595	MN146024	329,611	328,403	1209	346
Agr2	12839	MN146025	55,035	56,437	1403	389
Agr3	13190	MN146026	106,456	107,896	1441	358
Agr4	09164	MN146027	405,253	406,500	1248	342
Agr5	13291	MN146028	439,057	437,487	1571	430
Agr6	04120	MN146029	11,372	10,043	1330	346
Agr7	10454	MN146030	18,741	17,315	1427	387
Agr8	04444	MN146031	1,035,120	1,033,830	1291	353
Agr9	06743	MN146032	231,813	233,188	1376	372
Agr10	09008	MN146033	349,082	347,841	1242	308
Agr11	05024	MN146034	112,812	111,488	1325	355

^a^ID refers to the annotated TPS gene (AAE3_ID) in the *A. aegerita* genome (https://www.thineslab.senckenberg.de/agrocybe_genome)

Although sesquiterpenoids are ubiquitous in basidiomycetes, only a few sesquiterpenes derived from basidiomycetes have been characterized, and we know little about most of their biosynthesis. Because the sesquiterpene biosynthetic pathway is relatively small, heterologous expression of the entire pathway in a suitable host strain is the preferred method to retrieve the biosynthetic product produced by the genome of basidiomycetes. In addition, sesquiterpene synthase and terpenoid modifying enzymes are the reasons for the diversity of sesquiterpenes. The method of exploring sesquiterpenes in basidiomycetes by mining enzyme genes also provides ideas for other fungal biosynthesis pathways.

Conversely, our team is also actively researching the related content of *G. lucidum* sesquiterpenes. Currently, we have successfully cloned 21 genes from *G. lucidum* and expressed them heterologously in *E. coli*. During this study, we discovered that *G. lucidum* sesquiterpene products contain a variety of active substances, of which the most valuable may be an anticancer substance. In the future, we plan to continue to study *G. lucidum* sesquiterpenes to enrich the biosynthesis of basidiomycetes.

### Biosynthesis of diterpenoids

**Fig. 6:**
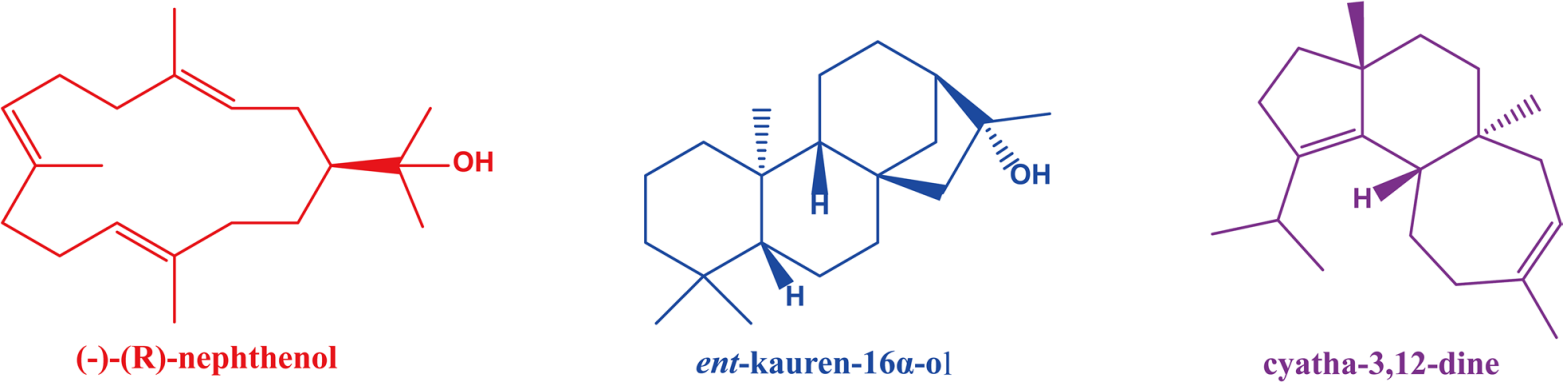
Three different diterpenoids produced by four di-TPS genes in basidiomycetes in *S. cerevisiae*

Diterpenoids are a variety of natural products derived from the C20 precursor geranylgeranyl pyrophosphate (GGPP), and more than 12,000 compounds have been described (Liu et al. 2020). Basidiomycetes are the primary abundant sources of diterpenoids. However, compared with the abundant diterpenoid synthases (di-TPSs) in ascomycetes, only three di-TPSs have been identified from basidiomycetes (Li et al. 2019). In recent years, the combination of genomic sequencing and synthetic biology techniques has enabled the rapid identification and characterization of di-TPSs from basidiomycetes fungi. In a previous study, 25 di-TPS genes were identified from the genomic data of 220 basidiomycetes by genomic data mining combined with the biosynthesis pathway of terpenes in *S. cerevisiae* and GC–MS analysis (Table 2). Four of them were functionally expressed in *S. cerevisiae* and produced three different diterpenoids (Table 3, Fig. 6), and the rest did not produce detectable compounds (Li et al. 2019). This study also provided new insight into the discovery of new diterpenoids from basidiomycetes based on genome data.

**Table 2:** Putative diterpene synthases (di-TPSs) from sequenced basidiomycete fungi and experimentally characterized fungal di-TPSs

Name	Protein ID	Species
Type I		
CopTC1	1742676	*Coprinellus micaceus*
CopTC2	645356	*Coprinellus pellucidus*
DenTC1	567829	*Dendrothele bispora*
DenTC2	818928	*Dendrothele bispora*
GymTC1	KIK55681.1	*Gymnopus luxurians*
GymTC2	KIK55687.1	*Gymnopus luxurians*
MarTC	956895	*Marasmius fiardii*
MonTC	KTB36256.1	*Moniliophthora roreri*
MycTC1	1209338	*Mycena galopus*
MycTC2	1989549	*Mycena haematopus*
SphTC1	KIJ41383.1	*Sphaerobolus stellatus*
SphTC2	KIJ46663.1	*Sphaerobolus stellatus*
SteTC1	XP_007305993.1	*Stereum hirsutum*
SteTC2	XP_007299393.1	*Stereum hirsutum*
UbiA type		
DenTC3	713581	*Dentipellis* sp.
DicTC	XP_007369786.1	*Dichomitus squalens*
GalTC	KDR74414.1	*Galerina marginata*
PanTC	1707524	*Panus rudis*
PenTC	KZV67417.1	*Peniophora* sp
RicTC1	2478742	*Rickenella fibula*
RicTC2	845642	*Rickenella mellea*
RicTC4	857243	*Rickenella mellea*
RicTC3	782838	*Rickenella mellea*

**Table 3:** Four basidiomycete genes that can produce di-TPS in *S. cerevisiae*

Gene	Location	Protein ID	Predicted functions	Species
*DenTC3*	scaffold_9:787690–789069	713581	UbiA-type Terpene cyclase	*Dentipellis* sp
*SteTC1*	XP_007305993.1	122776	Terpene cyclase	*S. hirsutum*
*PunTC*	scaffold_5:1536826–1540005	101867	Terpene cyclase	*P. strigosozonata*
*SerTC*	scaffold_3:2915485–2918654	413567	Terpene cyclase	*S. lacrymans*

Pleuromutilin, a typical diterpenoid compound of basidiomycetes, is used as the precursor of antibiotics (Lemke et al. 2020) and primarily inhibits the growth of gram-positive bacteria (GPPs) (Murphy et al. 2017). Due to a lack of hosts for heterologous expression of basidiomycete genes, the development of reliable expression systems is essential for genomic mining of natural products. To date, an experimental study has shown the heterologous expression of the pleuromutilin gene in *E. coli* (Xu et al. 2018). To develop a universal host for basidiomycete genes, gene expression was detected using the genomic DNA sequence of an ascomycete host (*Aspergillus oryzae*), and the fungal natural product biosynthesis gene was expressed directly from the genomic DNA of the system. As a result, 29 biologically active pleuromutilin sesquiterpene synthase genes and diterpenoid biosynthesis genes were successfully expressed using the system (Hu et al. 2019a, b). Because an increasing number of derivatives of diterpenoid antibiotics are produced biologically, understanding the biosynthetic pathway of pleuromutilin is a basic requirement for the large-scale production of natural products. To date, the biosynthetic pathway of pleuromutilin has been inferred only with limited experiments with isotopically labelled predicted precursors; however, the catalytic enzyme features and mechanisms involved in this biosynthetic pathway are still lacking (Alberti et al. 2017a). In the future, the combination of synthetic chemistry and synthetic biology will highlight a pathway to develop new diterpene derivatives.

*Hericium erinaceus*, a traditional medicinal fungus of basidiomycetes, is considered to have anti-dementia effects (Tsai-Teng et al. 2016), and the terpene compound hericin is considered to be the primary active ingredient in *H. erinaceus*. In recent years, the first genome and transcript sequence of *H. erinaceus* have been reported, with a genome size of 39.35 Mb and containing 9895 gene models. Scientists have found 12 genes that are responsible for the synthesis of terpene skeletons from KEGG pathway data and found that the gene encoding farnesyl pyrophosphate synthase (HerlA4642) was upregulated in the mycelium, with a change of more than 4.0 times compared with that in the fruit body. The transcription of acetyl-CoA acetyltransferase (HerlA4929) in the fruiting body stage was approximately sevenfold higher than that in the mycelium stage. Concurrently, the gene clusters involved in secondary metabolism were predicted, and it was confirmed that GGPPs (Her1A5912) were responsible for the biosynthesis of the diterpenoid precursor GGPP. In addition, two cytochrome P450 enzymes (Her1A5908 and Her1A5910) in the fruiting body were upregulated more than 20-fold compared to the mycelium in *H. erinaceus,* and sequencing analysis of the genome of *H. erinaceus* showed that genes involved in diterpenoid synthesis had different expression levels in the mycelia and fruiting bodies (Chen et al. 2017).

With the development of metabolic engineering and synthetic biology technology, based on optimizing diterpene active molecules and analysis of their biosynthetic pathways, genome mining and pathway optimization design of key elements are performed via pathway adaptation and environmental factor regulation in the natural host or high-efficiency production of the target product in a heterologous host. This process is a powerful method of developing new diterpenoids and applying them to medical treatment in the future.

### Biosynthesis of the triterpenoids

Triterpenes are a large class of natural products that are composed of six isoprene units, with complex and diverse chemical structures and extensive biological activities. Basidiomycetes are an important source for the discovery of triterpenoids. However, compared with plants, there are still few triterpenoid skeleton types that are found in basidiomycetes, and there is still much more research to be performed. With the rapid development of high-throughput sequencing technology and bioinformatics technology, some triterpenoid biosynthesis pathways with important biological activities have been gradually described, such as *P. cocos, Sanghuangporus baumii, Hypholoma sublateritium, Antrodia cinnamomea* and *G. lucidum*.

For example, *S. baumii* produces low levels of triterpenoids, which inhibits their use in medical treatment. Therefore, it is a research focus to modify strains based on synthetic biology and genetic engineering strategies to increase the yield of metabolites. The triterpenoids in *S. baumii* are useful medicinal ingredients, but the genes encoding the enzymes responsible for the isoprenoid biosynthetic pathway have poor characteristics. Therefore, scientists cloned the cDNA and promoter region of farnesyl diphosphate synthase (FPS) from *S. baumii* and performed bioinformatics analysis. Then, FPS was expressed in *E. coli* BL21. In addition, FPS expression levels were measured at different developmental stages of the hyphae. Compared with the 9-day hyphal control, the expression in the 11- and 13-day hyphal groups was upregulated by 49.3 times and 125.4 times, respectively. Through analysis, the triterpene content of *S. baumii* was found to be significantly correlated with the expression level of FPS at different developmental stages (Wang et al. 2020a, b, c). The results of this experiment showed that FPS genes are involved in regulating the biosynthesis of triterpenoids. In addition, reports have confirmed that the basidiomycete *H. sublateritium* can produce triterpenoid antitumour compounds. Basidiomycetes are difficult to operate at the molecular level. Scientists cloned the squalene epoxidase gene (erg1) from *H. sublateritium* and realized the genetic modification of *H. sublateritium* through *Agrobacterium tumefaciens*-mediated transformation. In this experiment, the gdhA promoter of *Agaricus bisporus* was coupled to the erg1 gene. Overexpression of the erg1 gene resulted in an increase in the production of clavaric acid by 32% to 97%, confirming that egr1 was involved in the biosynthesis of this antitumour product (Godio et al. 2007). The results of this experiment showed that squalene oxide seems to be the branch point of primary and secondary metabolites of basidiomycetes.

Previous studies on triterpenoids have primarily focused on the extraction and purification process, but the relationship between the content of triterpenoids and the expression levels of key genes has not been fully explored. This type of research may provide useful information for studying the function of key genes and ultimately increasing the production of triterpenoids. In addition, the most extensive research on triterpenoids has focused on *G. lucidum*. In the biosynthetic pathway of *G. lucidum*, a large number of key enzyme genes are involved in the production of *G. lucidum* triterpenoids.

*Ganoderma lucidum*, a fungus of *Polyporaceae* of basidiomycetes, has been used as a drug for more than 2000 years (Li et al. 2020; Geng et al. 2020), with pharmacological activities including antibacterial, antiviral, antitumour, anti-HIV-1, antioxidative and cholesterol-reducing effects (Abate et al. 2020; Kang et al. 2015; Ahmad 2020; Xu et al. 2011; Vallavan et al. 2020; Meng et al. 2019; Bhat et al. 2019; Wang et al. 2019a, b). According to published statistics, the annual sales of *G. lucidum* products have exceeded 2.5 billion US dollars (Zhang et al. 2017a, b, c). Due to the large demand and potential profit associated with the medicinal applications of this fungus, the biosynthesis of active ingredients in *G. lucidum* has become an important issue.

The primary active components of *G. lucidum* are polysaccharides and *G. lucidum* triterpenes (GLTs), which have immune regulation and antitumour effects (Tian et al. 2021; Shao et al. 2020; Do et al. 2021; Zhang et al. 2021a, b, c; Bharadwaj et al. 2019). *G. lucidum* is the fungus with the most triterpenoids, and more than 250 triterpenoid compounds have been identified from this species. However, compared with plants, we know relatively little regarding the biosynthesis of triterpenoid compounds in basidiomycetes (Wang et al. 2020a, b, c). It has been determined that the primary terpene component in *G. lucidum* is ganoderic acid (GA) (Shao et al. 2020; Liang et al. 2019), and more than 170 types of GA have been isolated from *G. lucidum* (Wang et al. 2021). Notably, all triterpenoid compounds in *G. lucidum* are tetracyclic triterpenoids (Bhat et al. 2019), and the chemical structure of *G. lucidum* triterpenoid compounds is based on lanolin sterane. Lanolin is a metabolite of lanosterol, and lanosterol is formed by cyclization of squalene (Liang et al. 2019). In 2003, Shiao et al. (2003) confirmed via the isotope labelling method that the terpenes in *G. lucidum* were indeed synthesized through the MVA pathway. The biosynthetic pathway of triterpenoids has been preliminarily characterized. To increase the yield of GA, scientists have conducted a large number of studies on the key enzyme genes involved in its biosynthesis.

Scientists used succinate dehydrogenase B (SDHB) as a selectable marker for the first time to establish a homologous genetic transformation system for *G. lucidum*. The *Agrobacterium tumefaciens* transformation system was used to overexpress the HMGR gene. Results showed that the overexpression of HMGR doubled the content of GA and simultaneously increased the accumulation of squalene and lanosterol (Xu et al. 2012). To determine the role of MVD in the biosynthesis of GA, scientists have used homologous FPS to overexpress GA, which effectively increases the production of GA but also results in increased expression of squalene synthase (SQS) and lanosterol synthase (LS). The SQS and LS transcription levels were upregulated 2.28- and 1.73-fold, respectively (Fei et al. 2019). In addition, *Agrobacterium tumefaciens* was used to mediate the transformation of *G. lucidum*, and Gl-MVD overexpression transformants were screened by PCR. The data showed that the transcripts were all overexpressed, and their triterpene content increased by approximately 17–101.4% (Shi et al. 2012). These results highlighted that MVD plays a key role in the biosynthesis of GA.

Squalene epoxidase is a type of biocatalyst. To study its effect on the biosynthetic pathway of GA, the SE gene was cloned from *G. lucidum* and overexpressed. The study found that SE was overexpressed, and the content of GA produced by the strain was two times that of the wild-type strain (Zhang et al. 2017a, b, c). This result indicates that the SE gene stimulates the biosynthesis of GA. In this study, SE and HMGR genes were simultaneously and strongly expressed. The results of the experiment found that the co-expressed strain had a higher acid content than the GA content of the single expressed strain (Zhang et al. 2017a, b, c), which proved that the joint co-expression of the two genes promoted GA biosynthesis. This research provides an effective basis for the biosynthesis of GA.

Lanosterol synthase (LS) is at the second branch point of the MVA synthesis pathway and is also a key enzyme. LS can catalyse the cyclization of 2,3-oxidized squalene to lanosterol. Additionally, in the liquid medium of *G. lucidum*, the overexpression of LS increased the content of GA. In the transgenic strains, the contents of lanosterol and ergosterol increased by 2.3- and 1.4-fold, respectively (Zhang et al. 2017a, b, c). The results of this experiment show that the LS gene can promote the biosynthesis of GA. In summary, the key enzyme genes in the biosynthesis pathway of GA strongly affect the yield of GA, but the mechanism underlying this phenomenon must be explored in more detail.

In recent years, a large number of terpenoids have been obtained from basidiomycetes, among which triterpenoids have been reported the most, and relatively few are sesquiterpenes and diterpenes. Because the terpenoids of basidiomycetes have extremely high medical value, better use of them will be an important research topic. By improving basidiomycete identification methods, culture methods, genetic engineering and other technologies, the terpenoid compounds of basidiomycetes can be developed and used more effectively, and the development of the medical industry can be promoted.

### Cytochrome P450 enzymes in basidiomycete terpenoids

Cytochrome P450 enzymes (CYP450) are important enzymes for secondary metabolism in plants and play an important role in synthetic biology (Mao et al. 2020). According to prior reports, more than 95% of the biosynthetic pathways of terpenoids go through one or more CYP catalytic steps (Xiao and Zhong 2016). Unknown cytochrome P450s (CYPs) in the biosynthetic pathway of terpenoids make heterologous production of related terpenoids difficult, and the slow development of some known CYPs markedly limits the efficiency of terpenoid biosynthesis (Xiao et al. 2019). According to reports, the CYP450 family modifies more than 97% of terpenoids. Different CYPs lead to structural diversity and different biological activities (Guo et al. 2016). The large-scale differentiation of basidiomycetes P450s has been shown from the analysis of the entire genome sequence of basidiomycetes. These data indicate that the molecular and functional diversity of P450s is the basis of the metabolic diversity of basidiomycetes (Ichinose 2013). Therefore, speeding up the discovery and design of terpenoid biosynthetic CYPs to fully achieve their huge industrial application potential has become the focus of modern research.

### CYP450 catalyses the biosynthesis of triterpenoids

Studies have identified candidate genes that encode biologically active compounds from *A. cinnamomea*. AcCyp51 encodes cytochrome P450 sterol 14-α-demethylase cloned from *A. cinnamomea*. AcCyp51 has been shown to be synthesized via the MVA pathway, cyclizing squalene into a lanostane triterpene skeleton, followed by demethylation to ergostane and modification to various triterpenoids, including antcins. Concurrently, the expression level of AcCyp5 in fruiting bodies was higher than that in other tissues. Uniquely, the five genes encoding the CYP512 P450 enzyme and the three genes encoding the CYP5140 enzyme all showed the highest expression in the fruiting body (Lu et al. 2014). Therefore, these genes may participate in the modification of ergostane-type triterpenoids.

**Fig. 7:**
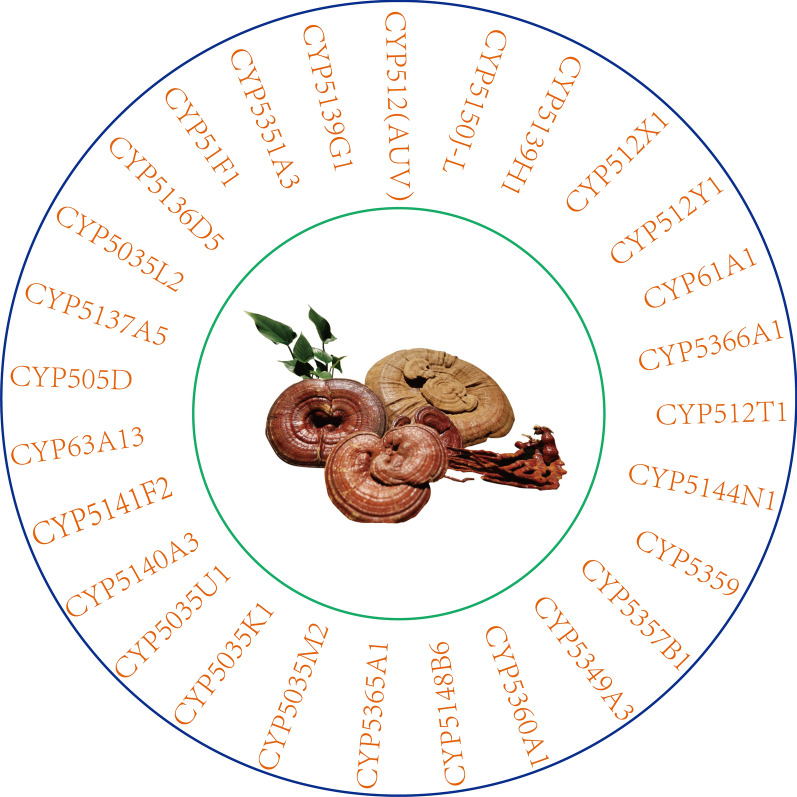
New family of cytochrome P450 enzymes unique to *G. lucidum*

There is a huge CYP450 family in the basidiomycetes *G. lucidum*. A total of 197 CYP genes are expressed in *G. lucidum*, of which 78 genes are upregulated during the transformation of mycelium to primordium. Analysis of expression profiles showed that these genes were positively correlated with the content of triterpenoids during development, suggesting that these 78 genes might be involved in the biosynthesis of triterpenoids from *G. lucidum*, and 28 of these genes were identified as members of a new family specific to *G. lucidum* (Chen et al. 2012) (Fig. 7). In the GA biosynthesis pathway, lanosterol is an important active precursor. To synthesize different GAs, lanosterol must be catalysed and modified by different cytochrome P450 enzymes to participate in the biosynthesis of GA (Xiao and Zhong 2016).

**Fig. 8:**
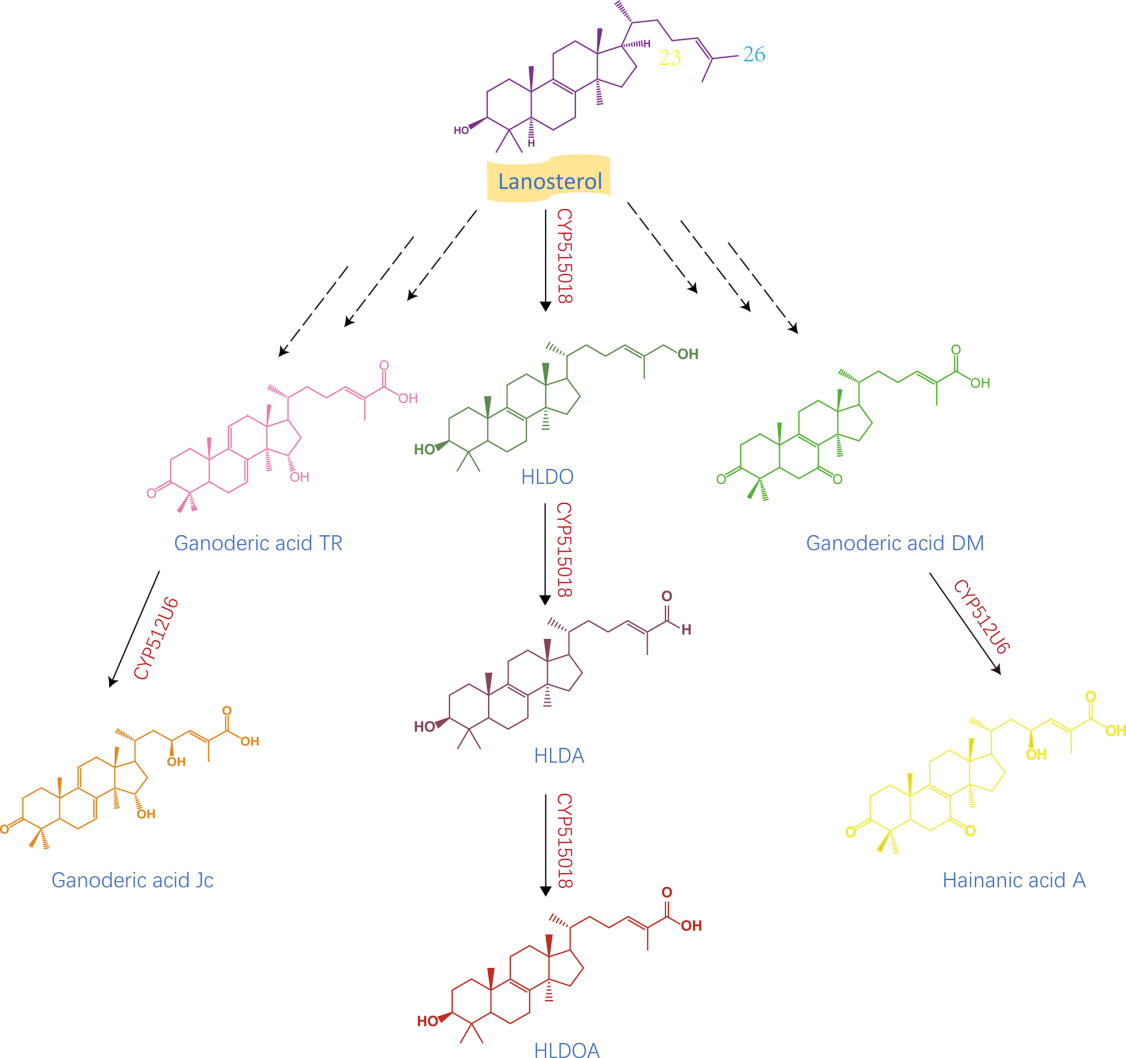
The C23 and C26 positions of lanosterol were catalyzed to produce HLDOA, hainanic acid A and GAJc

According to previous reports, 72 CYPs in *G. lucidum* were screened using the high-yielding lanosterol *S. cerevisiae* strain as the heterologous host, and results showed that CYP5150L8 could catalyse lanosterol to generate HLDOA through a three-step oxidation reaction at C-26 (Fig. 8). This report is the first on the heterologous synthesis of GA (Wang et al. 2018). Next, according to a previous report, a double expression system of CYP5150L8 and *G. lucidum* CYP450 reductase was constructed, and their conditions were optimized. By studying the fermentation capacity of the strain under optimal conditions, we found that the GA yield in the strain was 10 times higher than that reported previously (Lan et al. 2019). This study provided a reference basis for the high yield of GA.

According to prior reports, CYP512U6 plays an important role in the biosynthesis of GA. The GA CYP512U6 and GLCPR genes cloned from *G. lucidum* were expressed in the *S. cerevisiae* recombinant strain and analysed and compared by HPLC/HR-ESMS and HNMR. Results showed that CYP512U6 could hydroxylate GADM and TR at the C-23 position to generate Hainanic A and GAJc (Fig. 8), respectively. In this study, the microsomal portion of the yeast recombinant strain was incubated with GADM, and the resulting compound was designated GAZXYL after purification and identification. The CYP gene was also cloned and sequenced. Among the 78 CYP genes to be analysed, CYP512U6 showed the second largest transcriptional upregulation from hyphae to primordia (Yang et al. 2018). These results indicated that CYP512U6 was primarily responsible for the hydroxylation of oxygen-containing lanostane-type triterpenoids at the C-23 position.

Entire-genome sequencing of fungi, particularly basidiomycetes, describes the characteristic existence of ultra-large P450 genes, second only to those of plants (Syed et al. 2013). *G. lucidum* is the fungus with the most CYP genes. Through entire-genome sequencing analysis of *G. lucidum*, a total of 219 CYP genes (197 functional genes and 22 pseudogenes) were identified in *G. lucidum* (Chen et al. 2012). However, little is known regarding the CYP gene involved in terpene biosynthesis in *G. lucidum*, which is a major difficulty facing the field of *G. lucidum* research.

### Analysis of CYP450 research prospect

The currently discovered but unidentified CYPs are critical and challenging for efficient biosynthesis of terpenes. Early research on CYPs focused on gene deletion, gene silencing and in vitro enzyme determination, which largely relied on the genetic manipulation of the natural host, high CYP enzyme activity and the availability of substrates or precursors (Xiao et al. 2019). These limitations make the discovery of key CYPs difficult and unpredictable. In the future, microenvironmental engineering methods, such as improved electron transfer coupling; subcellular compartment targeting and engineering; and multienzyme complex engineering (Lee et al. 2018; Heng et al. 2018), may be required to reconstruct and increase the activity of CYP in heterologous hosts, thereby accelerating CYP discovery and enhancing terpenoid biosynthesis effectiveness.

White-rot fungi are basidiomycetes that can degrade a variety of aromatic compounds. *Phanerochaete chrysosporium* is the most widely studied white-rot fungus in terms of lignin decomposition and xenobiotic metabolism (Chang-Young et al. 2017). The genetic diversity of fungal P450 has been demonstrated by genome-wide sequencing, with up to 149 P450 genes identified in the *P. chrysosporium* genome (Matsuzaki and Wariishi 2004). However, there are few reports on its functions. Considering that it is not easy to perform simultaneous expression analysis for each of 149 P450 genes using conventional procedures, a team has developed a custom-designed 70-mer oligonucleotide-based microarray to study the genome-wide expression profile of P450. This design provides greater flexibility, thereby providing a higher level of hybridization specificity. The CYP450 genes were expressed in two different media (nutrient-rich and nutrient-limited media), and results showed that all 149 genes were expressed. Among them, 27 CYP450 genes are highly expressed. In the high expression group, 23 P450 genes were upregulated in nutrient-rich medium (two- to ninefold), while four genes were upregulated in nutrient-deficient medium (2- to 20-fold). Analysing the experimental results, it has been speculated that p450 is related to the degradation of exogenous compounds under eutrophic culture conditions (Syed and Yadav 2012).

Experiments have shown that the cytochrome P450 CYP5136A1 and CYP5136A3 in *P. chrysosporium* can catalyse the oxidation reaction of a variety of exogenous compounds. Scientists developed a heterologous expression system for CYP5136A1 and CYP5136A3 using the T7 RNA polymerase/promoter system in *E. coli*. By modifying and optimizing the N-terminal amino acid sequence of recombinant P450, the expression level of recombinant P450 was significantly improved. By coexpressing CYP5136A1 and the redox partner NADPH-dependent P450 reductase (CPR), the CYP5136A1 reaction system was reconstructed in *E. coli* entire cells, and results showed that the catalytic activity of CYP5136A1 was significantly increased (Hatakeyama et al. 2016). This result means that CYP5136A1 and CPR plays an important role in the heterologous metabolism of fungi. In recent years, *P. chrysosporium* has been heterologously expressed in yeast, and two CYPs (CYP5037B3 and CYP5147A3) in *P. chrysosporium* were identified as the primary isozymes involved in the metabolism of three neonicotinoids (NEOs), which have been widely used as botanical insecticides (Mori et al. 2021). In addition, CYP505D6 in *P. chrysosporium* has also been studied. The discovery that CYP505D6 can be used as a unique broad-spectrum substrate will make it an attractive candidate enzyme for the biotechnology industry (Sakai et al. 2018).

In recent years, it has become a mainstream trend to use a series of genome projects to conduct “omics” research to increase the understanding and advanced applications of the entire genome of basidiomycetes. An in-depth understanding of the sequence–structure–function relationship of p450 is a challenging task, and experimental screening is essential to elucidate the catalytic potential of p450. In particular, there are few studies on the biosynthesis of basidiomycetes p450s, which makes it difficult to predict their functions based on sequence homology. In this case, the development of rapid functional screening systems will open the door for future research on basidiomycetes p450.

## Conclusions

Basidiomycetes are rich sources of terpenes with extensive pharmacological activities, such as the antidepressant effect of *Armillaria mellea* (Zhang et al. 2021a, b, c), the anticancer and anti-inflammatory activity of *Antrodia camphorata* (Wang et al. 2019a, b), the antitumour and immunoregulatory effects of *L. rhinocerotis*, and the antitumour, anti-inflammatory and antiallergic effects of *Agaricus blazei*, *H. erinaceus* and *Grifola frontosa* (Lau et al. 2015). However, genetic manipulation of basidiomycetes is known to be difficult and immature. Recent genomic data and bioinformatics analysis have shown that fungi have a large number of biosynthetic gene clusters of bioactive natural products, but more than 90% are silent. Basidiomycetes are also high-yielding producers of natural products with diverse structures and biological activities (Masuya et al. 2019). However, although basidiomycota have great potential in the discovery of natural products, this field of drug discovery is basically unexplored compared with that of Ascomycetes.

In recent years, the development of synthetic biotechnology has benefited from the continuous advancement of gene sequencing technology, genetic engineering and protein engineering, and it has also profoundly affected traditional biotechnology, such as microbial molecular breeding. The “Synthetic Yeast Genome (SC 2.0) Project”, led by Professor JefBoeke of New York University, uses artificial design and synthetic genome technology to screen strains with marked growth advantages under different fermentation conditions, such as temperature and pH, and to develop practical production applications, such as food improvement and biofuel production (Sliva et al. 2015). Concurrently, the genome of microorganisms is minimized; nonessential genes are eliminated one by one, and only essential genes required for growth, replication, or synthesis of a certain product are retained as much as possible (Papizadeh et al. 2017; Mol et al. 2018). This method allows us to better understand the basic biochemical mechanisms of life and provides an important idea for selecting excellent strains as the basis for the synthesis of natural products.

Since 2010, heterologous fungal metabolite expression has been considered to be a powerful method for producing natural products (Oikawa 2020). Therefore, the ability to directly express basidiomycete biosynthesis genes in a suitable fungal host would markedly accelerate the functional characterization of the natural product pathway. Studies have focused on the discovery and study of STPS from the basidiomycete *Coniophora puteana*, heterologous expression in engineered *E. coli* to produce sesquiterpene compounds β-copaene and cubebol, which are industrially useful as food and flavour additives (Mischko et al. 2018). In addition, the ascomycete *A. oryzae* is an effective expression host for basidiomycete terpenoids, and the diterpenoid pleuromutilin and sesquiterpene synthase genes from *Clitopilus pseudopinsitus* have been successfully expressed in this system (Nagamine et al. 2019). Currently, the most commonly used hosts for heterologous expression are *E. coli* and yeast. In addition, filamentous fungi are typically selected when pursuing the complete biosynthesis of fungal secondary metabolites. These fungi typically have simple growth requirements and are suitable for large-scale fermentation. Among various fungi, *Aspergillus* is the most commonly used intermediate host. *Aspergillus nidulans* is a genetic model species in filamentous fungi and is also used as a heterologous host to study gene clusters of other species (Alberti et al. 2017a, b).

Although we have extensive knowledge of yeast and *E. coli*, not all pathways can be expressed well. Cell factory platforms that can operate under extreme temperatures, extreme pH values, and extreme salt concentrations may also be required. It is particularly time-consuming to develop a solid knowledge base for this new platform cell factory (Nielsen and Keasling 2016). Second, due to a lack of complex biological knowledge to regulate secondary metabolism, metabolic engineering cannot achieve the predictability reported in other projects. In native producers and heterologous hosts, attempts to manipulate regulatory genes in biosynthetic gene clusters have highlighted this knowledge gap (Teijaro et al. 2019). Therefore, strengthening knowledge acquisition and innovating new biotechnology is the current focus and a difficult task.

Through the exploration of core elements in synthetic biology, such as terpenoid synthesis pathways and the analysis of reasonable assembly methods, the establishment of predictive and regulatory metabolic pathways for organisms simultaneously combined with technologies such as genome synthesis, genome minimization, and genome editing can be used to create excellent chassis strains. This process will certainly promote the development of synthetic biology of terpenoid medicinal ingredients and lay the foundation for the construction of artificial cells to realize the large-scale production of medicinal terpenoids.

With the rapid development of biotechnology, an increasing number of new technologies have been applied to social production, research and development, and medical treatments. Gene engineering, mutation breeding and other techniques are widely used in animals and plants to obtain the required products or to achieve a specific purpose. In recent years, researchers have gradually begun to use gene engineering technology to improve the synthetic pathway of terpenoids, but it is less commonly used in higher fungi. The application of genetic engineering to basidiomycetes is a long process. Due to the increasing demand for terpenoid products in the social market and the unclear functions of many key enzyme genes, the rapid development of the fungi industry may be truly realized by accelerating relevant research and synthesizing excellent engineering strains with high yields.

## Data Availability

Not applicable.
